# *In vivo* susceptibility of *Plasmodium falciparum* to artesunate in Binh Phuoc Province, Vietnam

**DOI:** 10.1186/1475-2875-11-355

**Published:** 2012-10-26

**Authors:** Tran Tinh Hien, Nguyen Thanh Thuy-Nhien, Nguyen Hoan Phu, Maciej F Boni, Ngo Viet Thanh, Nguyen Thuy Nha-Ca, Le Hong Thai, Cao Quang Thai, Pham Van Toi, Phung Duc Thuan, Le Thanh Long, Le Thanh Dong, Laura Merson, Christiane Dolecek, Kasia Stepniewska, Pascal Ringwald, Nicholas J White, Jeremy Farrar, Marcel Wolbers

**Affiliations:** 1 Wellcome Trust Major Overseas Programme (MOP), Oxford University Clinical Research Unit (OUCRU), Ho Chi Minh City, Vietnam; 2 Hospital for Tropical Diseases, Ho Chi Minh City, Vietnam; 3 Institute of Malariology, Parasitology, and Entomology (IMPE), Ho Chi Minh City, Vietnam; 4 Phuoc Long Hospital, Binh Phuoc Province, Vietnam; 5 Faculty of Tropical Medicine, Mahidol University, Bangkok, Thailand; 6 Centre for Tropical Medicine, Nuffield Department of Medicine, University of Oxford, Oxford, UK; 7 Drug Resistance and Containment, Global Malaria Programme, World Health Organization, Geneva, Switzerland; 8 World Wide Anti-malarial Resistance Network (WWARN), Centre for Tropical Medicine, Nuffield Department of Medicine, Oxford University, Oxford, UK

**Keywords:** *Plasmodium falciparum*, Artesunate, Parasite clearance half-life, Parasite reduction ratio, Parasite clearance of >72 hours

## Abstract

**Background:**

By 2009, there were worrying signs from western Cambodia that parasitological responses to artesunate-containing treatment regimens for uncomplicated *Plasmodium falciparum* malaria were slower than elsewhere which suggested the emergence of artemisinin resistance. Vietnam shares a long land border with Cambodia with a large number of migrants crossing it on a daily basis. Therefore, there is an urgent need to investigate whether there is any evidence of a change in the parasitological response to the artemisinin derivatives in Vietnam.

**Methods:**

From August 2010 to May 2011, a randomized controlled clinical trial in uncomplicated *falciparum* malaria was conducted to compare two doses of artesunate (AS) (2mg/kg/day versus 4 mg/kg/day for three days) followed by dihydroartemisinin-piperaquine (DHA-PPQ) and a control arm of DHA-PPQ. The goal was characterization of the current efficacy of artesunate in southern Vietnam. The primary endpoint of this study was the parasite clearance half-life; secondary endpoints included the parasite reduction ratios at 24 and 48 hours and the parasite clearance time.

**Results:**

166 patients were recruited into the study. The median parasite clearance half-lives were 3.54 (AS 2mg/kg), 2.72 (AS 4mg/kg), and 2.98 hours (DHA-PPQ) (p=0.19). The median parasite-reduction ratio at 24 hours was 48 in the AS 2mg/kg group compared with 212 and 113 in the other two groups, respectively (p=0.02). The proportions of patients with a parasite clearance time of >72 hours for AS 2mg/kg, AS 4mg/kg and DHA-PPQ were 27%, 27%, and 22%, respectively. Early treatment failure occurred in two (4%) and late clinical failure occurred in one (2%) of the 55 patients in the AS 2mg/kg group, as compared with none in the other two study arms. The PCR-corrected adequate clinical and parasitological response (APCR) rates in the three groups were 94%, 100%, and 100% (p=0.04).

**Conclusions:**

This study demonstrated faster *P*. *falciparum* parasite clearance in southern Vietnam than in western Cambodia but slower clearance in comparison with historical data from Vietnam. Further studies to determine whether this represents the emergence of artemisinin resistance in this area are needed. Currently, the therapeutic response to DHA-PPQ remains satisfactory in southern Vietnam.

**Trial registration:**

NTC01165372

## Background

Malaria remains a public health challenge in Vietnam despite a substantial reduction in the incidence of disease over the last 20 years. In 2011 there were 45,588 cases reported to the National Malaria Control Programme (Annual Report NMCP 2011) and 14 deaths attributed to malaria. Vietnam was one of the first countries to deploy artemisinin derivatives in its fight against malaria over twenty years ago [1]. The Vietnamese health authority took the decision to deploy these drugs because of the rising morbidity and mortality from malaria and the high level of drug resistance to chloroquine, sulphadoxine-pyrimethamine, and mefloquine. Since 2005, Vietnam has used dihydroartemisinin-piperaquine (DHA-PPQ) as the first-line treatment for *falciparum* malaria [2]. By 2009, there were worrying signs from western Cambodia that parasitological responses to artesunate-containing treatment regimens for uncomplicated *falciparum* malaria were slower than elsewhere and that unusually high failure rates with artesunate–mefloquine had been reported [3-5]; both of these signals suggested the emergence of artemisinin resistance. In addition, prolongation of parasite clearance time (PCT) has been reported on the Thai-Myanmar border [6] and evidence for artemisinin resistance has recently been reported from this area [7], indicating that artemisinin resistance may be spreading outside Cambodia. Vietnam shares a long land border with Cambodia with a large number of migrants and laborers crossing it on a daily basis. Therefore, there is an urgent need to investigate whether there is any evidence of a change in the parasitological response to the artemisinin derivatives in Vietnam. A randomized controlled trial in uncomplicated *falciparum* malaria was conducted, to compare two doses of artesunate (AS) followed by dihydroartemisinin-piperaquine (DHA-PPQ) with a control arm of DHA-PPQ, to characterize the current efficacy of artesunate in a malaria endemic area of southern Vietnam along the Cambodian border.

## Methods

### General consideration and justification of intervention

The primary aim of this study was to find out if there was prolonged parasite clearance in patients receiving artesunate mono-therapy in Vietnam. Preliminary results from routine monitoring of DHA-PPQ effectiveness performed by the Vietnam Malaria Control Program in the Phuoc Long area had suggested a high proportion of positive blood smears on day 3 (18%) (LT Dong, unpublished data). Thus, there was an urgent need for such a trial.

WHO recommendations on how to detect resistance to artemisinins was applied [8]: conducting controlled research studies with oral artesunate monotherapy and monitoring the parasite clearance half-life as well as the proportion of patients who are parasitaemic on day 3 (72 hours) as indicators of delayed parasite clearance. If this proportion exceeds the “rule-out threshold” of 10%, further detailed studies (pharmacokinetic and genetic) are advised to confirm the presence of artemisinin resistance in the area. Failure to clear parasites by day 3 indicates a change in the pattern of parasite susceptibility to artemisinins and is probably the first stage of artemisinin resistance, which is thought to be associated with a loss of activity against the early ring stages [3]. It was not deemed ethical to treat patients with monotherapy throughout their treatment course and the chosen treatment schedule was initial artesunate monotherapy to assess early parasite clearance followed by three days of DHA-PPQ according to national policy in Vietnam. The current recommended dose of artesunate in Vietnam is 4 mg/kg on day 1 and 2 mg/kg on days 2 and 3. However, this dose has been criticized as being insufficient to clear parasites. Therefore, two different artesunate regimens with different daily doses of 2 vs. 4 mg/kg/day were studied for easier comparison with data from western Cambodia [3]. We also included DHA-PPQ arm as a control arm. Higher DHA-PPQ fixed - dose combination than the current regimen was used because this could give an advantage in parasite clearance. In addition, a three-tablet regimen (once a day for 3 days) may be more convenient for patients than the current regimen (four tablets bid for the 1st day, then two tablets once per day). Randomizing patients to three different treatments also allowed us to perform formal comparisons between the three regimens, an important additional aim of this study.

### Study site and participants

The study was conducted during the malaria transmission season, from August 2010 to May 2011 at Phuoc Long District Hospital (150 beds), Binh Phuoc Province. Phuoc Long District, 170 km northeast of Ho Chi Minh City, is an elevated forested area of Binh Phuoc Province. Phuoc Long covers 1857 sq km in area, is divided into 21 communes and towns, and is home to a population of 197,897 including an ethnic minority population of 31,918. Phuoc Long is considered as the epicenter of malaria of Binh Phuoc province and the southern provinces of Vietnam. In 2009, there were 251 cases of confirmed malaria admitted to this District Hospital, including 138 cases of *Plasmodium falciparum*. The study population consisted of patients with uncomplicated *P*. *falciparum* malaria as detailed below. All adult patients (≥15 years old) gave written informed consent before participation. Parents or guardians gave informed consent on behalf of children. Children aged 12 years or older additionally gave assent to participate.

### Inclusion criteria

Consecutive patients attending Phuoc Long District Hospital or a nearby community health station were recruited into the trial if they fulfilled the following criteria: (a) male and aged > 10 years or female and aged between >10 and <12 years old, provided they had not reached menarche; (b) mono-infection with *P*. *falciparum* detected by microscopy; (c) asexual parasitaemia of 10,000–100,000/μL; (d) axillary or tympanic temperature ≥ 37.5°C or history of fever during the previous 24 h; (e) ability to swallow oral medication; (f) ability and willingness to comply with the study protocol for the duration of the study and to comply with the study visit schedule; and (g) informed consent/assent given as stated above. The WHO instruction to exclude pregnant women in the first three months was applied because artemisinin is contra-indicated during the first trimester. As the procedure to identify pregnancy with a urine test and counseling for female patients who are in child bearing age were too complicated for this field trial the decision to not include women who menstruate or are aged over 12 years was made to guarantee no pregnant women in this study. The range of asexual parasitaemia from 10,000–100,000/μL was chosen based on results of the paper by Stepniewska *et al*. [9] which suggested that the proportion of positive blood smears on day 3 would be a tool for assessing population levels of artemisinin resistance and artemisinin resistance is highly unlikely if the proportion of patients with a positive smear on day 3 (72 hours) is <3% among patients with parasite densities of >10,000 to <100,000 parasites/μL at enrolment. Another reason was to aid comparisons with other studies in the region. Additionally, according to National Guidelines in Vietnam high parasitaemia >100,000/μL is an indicator of potential severe malaria in Vietnam and should be treated with intravenous therapy.

### Exclusion criteria

Patients were excluded if they had one or more of the following conditions (a) presence of general danger signs or severe *falciparum* malaria according to the definitions of WHO 2009; (b) mixed or mono-infection with another *Plasmodium* species detected by microscopy; (c) presence of severe malnutrition (defined as a child whose growth standard is below −3 z-score, has symmetrical edema involving at least the feet, or has a mid-upper arm circumference < 110 mm); (d) presence of febrile conditions due to diseases other than malaria (eg measles, acute lower respiratory tract infection, severe diarrhea with dehydration) or other known underlying chronic or severe diseases (eg cardiac, renal and hepatic diseases, HIV/AIDS); (e) regular medication, which might interfere with anti-malarial pharmacokinetics; (f) receipt of anti-malarial drugs in the previous 48 hours; (g) history of hypersensitivity reactions or contraindications to any of the medicine(s) being tested or used as alternative treatment(s); or (h) previous splenectomy.

### Intervention

This study was an open-labelled, randomized controlled trial of the efficacy of artesunate (AS) and dihydroartemisinin-piperaquine (DHA-PPQ) in acute uncomplicated *falciparum* malaria with three arms: (i) AS 2mg/kg/day for three days, (ii) AS 4mg/kg/day for three days or (iii) DHA-PPQ (DHA 2.4 mg/kg/day + PPQ 19.2 mg/kg/day) for three days. Following randomization, the study research doctors were aware of treatment assignment. However, both the patients and nonmedical staff (nurses, microscopists, technicians and pharmacists) remained blinded to the treatment allocation. This trial was not primarily designed to compare the efficacy of study drugs *per se* but to determine if there is any evidence of prolonged parasite clearance following oral AS in *falciparum* malaria patients. The two AS arms were followed by a full course of DHA-PPQ (DHA 1.6 mg/kg/dose + PPQ 12.8 mg/kg/dose), which started on day 4: two doses on the first day, and a single daily dose on the second and third days (total three days therapy with DHA-PPQ) as per Vietnamese national treatment guidelines.

AS was sourced from Guilin Pharmaceutical Company, PRC, supplied by the WHO and DHA-PPQ was sourced as Artecan®, OPC from Vietnam distributed by the National Malaria Control Programme of Vietnam. All doses of medicine were administered and recorded under the supervision of qualified members of the staff designated by the principal investigator.

### Randomization

Block randomization with an allocation ratio of 1:1:1 and variable block lengths of six and nine were used to make the prediction of the randomized treatment assignment of patients impossible. The random allocations were placed in sealed opaque envelopes, which were kept in a locked drawer and opened by the research pharmacists once each patient was enrolled into the trial after satisfying the inclusion/exclusion criteria. Patients were enrolled in the order they presented and the sealed envelopes were opened in strict numerical sequence. In addition to the treatment arm assignment, patients were randomly allocated to one of two blood-sampling schemes for population pharmacokinetic analysis.

### Clinical and laboratory procedures

On admission patients were weighed, examined fully, and had baseline blood samples taken for full blood count and basic biochemistry. Blood was obtained by a finger-prick for haematocrit measurements and blood smears every six hours until two consecutive smears were negative for asexual stages of *P*. *falciparum*. Thick and thin blood films were examined on days 2, 3, 7, 14, 21, 28, 35 and 42 and on any other day if the patient returned spontaneously. The parasitaemia was determined from the number of parasitized red cells per 1,000 red cells (thin film) or the number of parasites per 400 white blood cells (WBC) (thick film). For each patient, all blood smears at baseline (100%), all negative smears (100%), approximately 10% of positive smears (randomly selected), and approximately 70% of smears with one parasite per 400WBC were read twice by two independent qualified microscopists. Parasite densities were calculated by taking the arithmetic mean of the two counts as recommended by WHO [10]. Blood smears with discordant microscopist results (differences in species diagnosis, discrepancies in the parasite density of > 50%, or disagreement on presence/absence of parasites) were re-examined by a third independent microscopist, and parasite densities were calculated by averaging the two closest counts. The study patients were observed for 30 min after drug administration for adverse reactions or vomiting. Any patient who vomited during this observation period was re-treated with the same dose of medicine and observed for an additional 30 min. If the patient vomited again, he or she was withdrawn and offered rescue therapy. Patients in all three arms were monitored for 42 days. The follow-up consisted of a fixed schedule of check-up visits on day 7, 14, 21, 28, 35 and 42 and corresponding clinical and laboratory examinations. Polymerase Chain Reaction (PCR) analysis was used to distinguish between true recrudescence due to treatment failure and re-infection. Genotyping was done with samples of all recurrent parasitaemia cases. Parasite DNA was extracted from dried blood spot samples by the Chelex extraction method [11]. Nested PCR was processed using specific primers for the three most common molecular markers *msp1*, *msp2* and *glurp*[12]. The results were analysed based on WHO definitions of recrudescence and new infection [13]. The study protocol, informed consent documents, relevant supporting information, and all types of patient recruitment or advertisement information were submitted to the Ethical Committee of the Vietnam Institute of Malariology, Parasitology and Entomology, the Oxford University Tropical Research Ethics Committee and the WHO Research Ethics Review Committee. The trial was registered at ClinicalTrials.gov (NCT01165372) [14].

### Outcome measures

The primary endpoints of this study was the parasite clearance rate constant K, i.e. the slope of the log parasitaemia–time linear relationship. An equivalent and more easily interpretable measure, the parasite clearance half-life T_1/2_ was also reported. T_1/2_ can be expressed as T_1/2_ = 0.693/K. The statistical models used to estimate the parasite clearance rate constant based on longitudinal parasite counts were fitted using the Parasite Clearance Estimator developed by the World-Wide Antimalarial Resistance Network (WWARN) [15]. Secondary endpoints included (i) PCR-uncorrected and corrected parasitological efficacy of the three treatment arms over 72 hours and during the follow-up period of 42 days according to WHO guidelines [10] classifying patients as early treatment failures, late clinical failures, late parasitological failures, or adequate clinical and parasitological responses (ACPR); (ii) Parasite clearance time (PCT100), defined as the time in hours from the first treatment dose to the first of two consecutive parasitaemia counts of zero (patients without documented parasitaemia clearance were censored at the time of the last measured positive parasite count); (iii) the proportion of patients with a parasite clearance time >72 hours after starting each treatment; (iv) parasite-reduction ratios at 24 and 48 hours, defined as the baseline parasitaemia value divided by the value at 24 or 48 hours, respectively; (v) fever clearance time, defined as the time in hours from the first treatment dose to the start of the first sustained period of 24 hours without fever (ie temperature<37.5°C);and (vi) adverse and serious adverse events.

### Sample size calculation

Parasite clearance half-lives calculated for 196 Vietnamese malaria patients enrolled in a clinical trial between 1996–2003 [16] had an approximate log-normal distribution with a median of 3.55 hours and a coefficient of variation of 0.55. Based on these assumptions, 50 patients in each treatment group provide 80% power (i) to detect a 0.75-fold difference in geometric mean half-lives between any two treatment groups (a 75% prolongation of parasite clearance half-life is what has been observed in the emergence of artemisinin resistance), and (ii) to show that the half-life in any treatment group is significantly different from 3.55 hours should the true slope in that group be 4.37 hours or larger. In order to accommodate a maximum loss-to-follow-up rate of 10%, the sample size was set to 165 patients to meet the total desired set of final endpoints from 150 patients.

### Statistical analysis

Continuous parasitological response measures were summarized as median (inter-quartile range) (IQR) and compared between the groups with the Kruskal-Wallis test. Parasite and fever clearance time were summarized with Kaplan-Meier estimates of median and inter-quartile range, and comparisons between groups were based on the log-rank test. If the overall comparison between groups was significant, pair-wise group comparisons were also performed. In accordance with WHO guidelines [10], the risk of treatment failure was calculated based on the Kaplan-Meier method including all randomized patients and, additionally, as a proportion for the subset of patients reaching documented failure or APCR. Comparisons between the groups were based on the log-rank test or Fisher’s exact test, respectively. All endpoints were reported and compared both on the intention-to-treat (ITT) population containing all randomized patients and the per protocol population which excluded all patients who were withdrawn or lost to follow-up during the first three days of therapy. All analyses were performed with the statistical software R2.14.1 (R Foundation for Statistical Computing, Vienna, Austria) and the companion R package mculst 4.0 [17] (for exploratory normal mixture modeling of log-transformed parasite clearance half lives).

## Results

**Figure 1:**
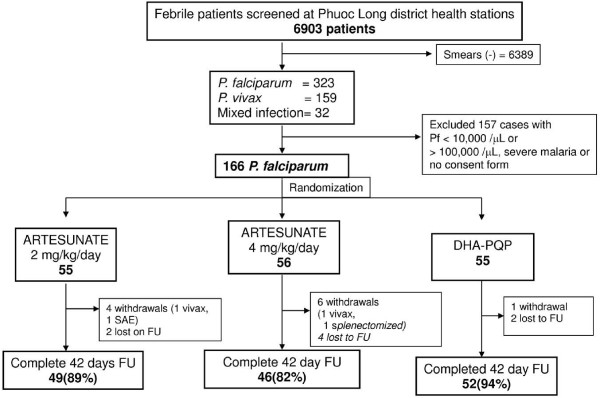
Study flow diagram.

The screening and recruitment of malaria patients are described in Figure 1. The proportion of patients with positive smears amongst those presenting to the health station was 514/6,903 (7%). Most patients recruited into the study (155/166 = 93%) were adults. On admission 153/166 (92%) patients were febrile and the others had symptoms of headache or fatigue with a history of fever. One patient in each treatment group refused to take anti-malarial drugs. One patient had *Plasmodium vivax* on their day two blood smear (AS 2mg/kg), one was splenectomized (AS 4mg/kg), and one was treated with IV artesunate (AS 2mg/kg, see adverse events below); all six were excluded from the per-protocol population. There were three, eight and two additional patients in the AS 2mg/kg, AS 4 mg/kg and DHA-PPQ arms, respectively, who withdrew from the study, were lost to follow-up, or had *P*. *vivax* on their blood smear after completion of therapy but before day 42. Therefore, 49, 46 and 52 patients in the three treatment groups completed the study follow-up schedule. The characteristics of the three treatment groups at study enrollment were similar (Table 1).

**Table 1:** Baseline characteristics by treatment group

**Characteristics**	**Artesunate 2mg/kg**		**Artesunate 4mg/kg**		**DHA-Piperaquine**	
	**n**	**(N=55)**	**n**	**(N=56)**	**n**	**(N=55)**
Age (years)	55	25 (20,38)	56	27 (21,35)	55	27 (21,33)
Children (< 15 years old)	55	4 (7%)	56	4 (7%)	55	3 (5%)
Sex	55		56		55	
Female		2 (4%)		2 (4%)		2 (4%)
Male		53 (96%)		54 (96%)		53 (96%)
Ethnicity	55		56		55	
- Kinh (Vietnamese)		26 (47%)		21 (38%)		19 (35%)
- S’tieng		25 (45%)		25 (45%)		26 (47%)
- Other ethnicities		4 (7%)		10 (18%)		10 (18%)
Previous malaria episodes	55	24 (44%)	55	25 (45%)	55	26 (47%)
Febrile on admission	55	55 (100%)	56	51 (91%)	55	47 (85%)
Temperature ( °C)	52	39.3(38.5,40.0)	56	38.6 (38.0,39.7)	55	39.0(37.9,39.5)
Height (cm)	55	164(160,169)	56	161(158,167)	55	163(158,167)
Weight (kg)	55	55(49,60)	56	53(50,58)	55	54(48,59)
Parasitaemia (parasites/μL)	55	27129 (12282,50868)	56	29192(19415,55892)	55	29390(16620,55578)
Hematocrit (%)	54	39.4(36.2,43.7)	55	40.0(37.4,42.9)	54	41.6(37.9,44.9)
Hemoglobin (g/dL)	53	13.8(12.6,15.4)	53	14.3(13.3,15.8)	51	14.2(12.9,16.0)
White Blood Cell (x 10^9^/L)	53	5.9(4.6,7.7)	54	5.6(4.4,7.2)	53	6.3(5.1,8.3)
Neutrophils (%)	50	78.7(66.5,82.9)	50	77.2(67.6,83.4)	48	74.9(67.2,80.6)
Lymphocytes (%)	54	15.8(11.7,26.6)	55	15.4(11.3,26.5)	54	19.1(13.6,24.6)
Platelets (x 10^9^/L)	50	93(69,140)	49	119(79,139)	49	111(70,154)
BUN (mmol/L)	52	17.1(14.5,18.2)	55	16.4(14.6,18.4)	54	16.6(14.3,19.1)
Serum creatinine (μmol/L)	53	79.6(61.9,88.4)	55	79.6(70.7,88.4)	54	79.6(70.7,88.4)
Total Bilirubin (μmol/L)	52	18.0(15.4,23.9)	50	20.5(13.4,27.0)	54	20.5(15.4,28.6)
Blood glucose (mmol/L)	52	4.9(4.3,6.4)	55	5.2(4.7,6.4)	54	5.2(4.4,6.1)
AST (IU/L)	53	35(27,49)	55	40(27,50)	54	40(30,58)
ALT (IU/L)	53	37(29,54)	55	39(29,64)	54	42(30,65)

Summary statistic is absolute count (%) for categorical variables and median (IQR) for continuous data.

### Parasitaemia-related endpoints and fever clearance time

**Figure 2:**
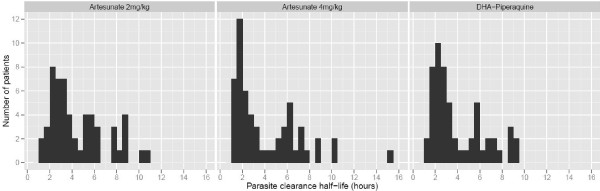
**Histograms of parasite clearance half**-**lives by treatment group.**

**Figure 3:**
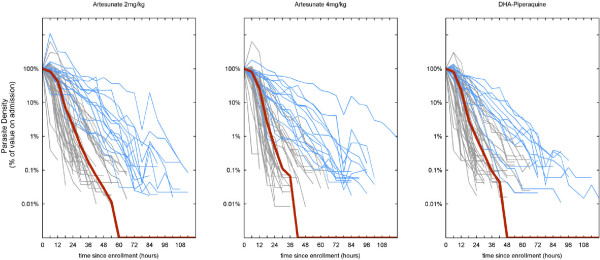
**Parasite clearance curves for all 166 patients in the study.** Median clearance times are shown in red. Patients who had >0.01% baseline parasitemia at 72 hours are shown in blue.

Endpoints are summarized in Table 2 (ITT population) and Additional file 1: Table S1 (per protocol population). Parasite clearance rate half-lives and parasite clearance times (PCT100) were similar across treatment groups (Figures 2 and 3). Notably, more than 20% of patients had a PCT100>72 hours in all three groups.

**Table 2:** **Summary of primary and secondary endpoints by treatment group** (**ITT population**)

**Characteristic**	**Artesunate 2mg/kg**	**Artesunate 4mg/kg**	**DHA-Piperaquine**	**Overall comparisons**
	(**N=55**)	(**N=56**)	(**N=55**)	(**p-value** )
Clearance rate constant (“slope”) K				
[ − log_e_ parasitaemia/hour] $				
- median (IQR)	0.20(0.12,0.27)	0.25(0.11,0.39)	0.23(0.12,0.33)	0.19
Clearance half life T_1/2_ [hours]				
- median (IQR)	3.54(2.61,5.88)	2.72(1.80,6.31)	2.98(2.10,5.82)	0.19
- geometric mean (95%CI)	3.85(3.32,4.46)	3.32(2.67,3.89)	3.38(2.89,3.95)	
-T_1/2_ > 6.2hours	11/55(20%)	15/55(27%)	10/55(18%)	
Treatment outcomes – WHO				
[PCR-uncorrected]				
- Early treatment failure (ETF)	2 (4%)	0 (0%)	0 (0%)	
- Late clinical failure (LTF)	1(2%) *	0 (0%)	0 (0%)	
- Late parasitological failure	0 (0%)	0 (0%)	1 (2%)£	
- Withdrawn or lost from follow-up	6 (11%)	10 (18%)	3 (5%)	
- Adequate clinical and parasitological response (ACPR)	**46** (84%)	**46** (82%)	**51** (93%)	
Risk of failure [PCR-uncorrected]				
- Kaplan-Meier analysis (ITT) #	6%	0%	2%	0.16
- Proportion (“per protocol”)	3/49 (6%)	0/46 (0%)	1/52 (2%)	0.21
Risk of failure [PCR-corrected]				
- Kaplan-Meier analysis (ITT) #	6%	0%	0%	0.04¥
- Proportion (“per protocol”)	3/49 (6%)	0/46 (0%)	0/51 (0%)	0.07
Parasite clearance time (PCT100)				
- median (IQR) [hours] #	60 (36, 78)	42 (30, 78)	48 (36, 72)	0.49
- Proportion with PCT>72h #	0.27	0.27	0.22	-
Parasite-reduction ratio (%) £				
- at 24 hours – median (IQR)	48 (5,282)	212 (11,2931)	113 (12,561)	0.02 €
- at 48 hours – median (IQR)	3430 (151,Inf)	Inf (217,Inf)	Inf (435,Inf)	-
Fever clearance time #				
- median (IQR) [hours]	30 (18, 42)	24 (12,42)	24 (18,36)	0.86

Summary measures in each group are median (IQR) or n (%).

($) Slope could not be estimated for one patient on Artesunate 4mg/kg, (*) PCR: recrudescence, (£) PCR: new infection, (#) Kaplan-Meier estimates, (£) Missing follow-up parasitaemia values at 24 and 48 hours replaced by 0 if the patient had prior documented parasite clearance; no missing data remained after this imputation. Values of infinity (Inf) correspond to patients without parasitaemia at 24 or 48 hours, respectively.

¥ Pairwise comparisons: Artesunate 2mg/kg vs. Artesunate 4mg/kg (p=0.08), Artesunate 2mg/kg vs. DHA-Piperaquine (p=0.07).

€ Pairwise comparisons: Artesunate 2mg/kg vs. Artesunate 4mg/kg (p=0.01), Artesunate 2mg/kg vs. DHA-Piperaquine (p=0.048), Artesunate 4mg/kg vs DHA-Piperaquine (p=0.27).

The Spearman rank correlation between initial parasite densities and PCT100 was 0.23 (p=0.003; cor=0.22 (AS 2mg/kg), 0.28 (AS 4mg/kg), and 0.22 (DHA-PPQ)). No significant rank correlation was observed between the initial parasite densities and parasite clearance half-lives (cor=0.03, p=0.67).

In the PCR-corrected analysis, no treatment failures were observed in the AS 4mg/kg and the DHA-PPQ groups compared to three (6%) treatment failures for patients receiving AS 2mg/kg (p=0.04 for the overall comparison among all three treatment groups). Similarly, parasite reduction ratios at 24 hours were significantly lower for patients receiving AS 2mg/kg (p=0.02). Eighteen out of 166 (11%) patients had detectable gametocytaemia at some time during the study period: on admission five (9%), six (11%) and four (7%) patients receiving AS 2mg/kg, AS 4mg/kg and DHA-PPQ, respectively, as well as two patients, one of each AS group (day 1) and one patient in the DHA-PPQ group (day 7). Gametocyte clearance times varied from six hours to 42 days. Fever clearance times were similar in all three groups (p=0.86).

Visually, the parasite clearance times in Figure 2 appear to be bimodal for all three arms. Indeed, exploratory log-normal mixture modeling of all parasite clearance half lives indicated that the best fitting model to explain their distribution is a mixture of two log-normal distributions. Specifically, this model suggests two underlying populations contributing 58% and 42% to the total population, and having geometric mean half-lives of 2.24 and 6.44 hours, respectively, and a common coefficient of variation of 0.32. Of note, this mixture model fitted the data much better than a single log-normal distribution with an improvement in the BIC (Bayesian information criterion) of 20.43. If real, a bimodal distribution of parasite clearance times would suggest that certain parasite sub-populations may be associated with longer or shorter clearance phenotypes. This would be consistent with the results of recent studies in Asia, [7][18][19] all of which show a strong genetic association with the clearance phenotype.

### Adverse and serious adverse events

There were 39 adverse events recorded: 14 (25%) in the AS 2mg/kg group, 12 (21%) in the AS 4mg/kg group and 13 (24%) in the DHA-PPQ group. Thirty-three of these events were low WBC counts (<5000. 10^9^ / L). All patients recovered uneventfully after two weeks. Only two serious adverse events were reported: one male patient in the AS 4mg/kg group was admitted to the hospital on day 21 because of dengue fever. The second case (AS 2mg/kg) was a 24- year old male patient with a parasitaemia of 90,800/ L at enrolment; six hours later the GCS dropped to 14/15 and parasitaemia increased to 116,008/ L. This patient was treated with IV artesunate because of deterioration of consciousness and was then given DHA-PPQ for 3 days as recommended by the Vietnam Malaria Control Program. His complete (100%) parasite clearance time was 60 hours but for determination of the treatment outcome (WHO definition), he was treated as withdrawn which was agreed to after reporting to the sponsor of the trial – the WHO. The patient made a full recovery, was discharged on day 6, and followed up until day 42 for safety reason.

There were two early treatment failures (both on AS 2m/kg). Both patients had positive smears on day 3 (+72hours) and were still febrile. At the end of their treatment course one patient had completely recovered and the other presented with a late parasitological failure at day 28 due to recrudescence.

## Discussion

This study was designed to identify cases of artemisinin-resistant *P*. *falciparum* malaria in southern Vietnam because of the increasing concern that artemisinin resistance has spread beyond western Cambodia [20]. To assess the real efficacy of artesunate – separate from the effects of its partner drug in artemisinin combination therapy (ACT) – the investigational treatments in this trial during the first three days were either 2 mg/kg/day or 4 mg/kg/day of artesunate alone, followed by a full dose of DHA-PPQ started on day 4 to guarantee the best anti-malarial treatment for patients and to comply with the recommendations of the National Malaria Control Programme of Vietnam. Artemisinin resistance is typically identified by prolongation in parasite clearance times [4] but in field studies, parasitaemia measures are often only performed once a day, typically on admission and on days 2 and/or 3 and day 7, in order to assess *in vivo* susceptibility of anti-malarial drugs. Once-daily parasitaemia measurements do not allow an accurate assessment of the parasite clearance times. To provide an accurate characterization, blood smears were taken every six hours in our study until two successive negative smears were observed. The median parasite clearance time was 60 hours (IQR: 36–78) for the AS 2mg/kg initial artesunate regimen, 42 hours (IQR: 30–78) for the 4 mg/kg regimen, and 48 hours (IQR: 36–72) for DHA-PPQ (P = 0.29). This is comparable with the results of the 2009 study from Wang Pha, western Thailand, where median parasite clearance times of 54 and 48 hours were reported but is considerably shorter than results from Pailin, Cambodia, where the median parasite clearance time was 84 hours (IQR: 54–96) for the 2 mg/kg dose and 72 hours (IQR: 60–96) for the 4 mg/kg dose [3].

An alternative measurement that has been used to assess parasite clearance is the parasite reduction ratio (PRR). For this study, these measures indicate that after 24 hours 97.93% (AS 2mg/kg), 99.51% (AS 4mg/kg), and 99.12% (DHA-PPQ) of parasites have been cleared from the blood. This corresponds to a 10^2^-fold reduction after 24 hours and a >10^4^-fold reduction after 48 hours (Table 2). These PRRs are comparable to those observed in Wang Pha Western, Thailand, and suggest faster clearance of parasites by approximately one order of magnitude than those reported from Pailin, Cambodia [3].

Recently, the slope of the log parasitaemia decline over time (or equivalently, the parasite clearance half-life) has been proposed as the most sensitive and robust measure for studying the anti-malarial effect of drugs [15] and therefore this was the designated primary endpoint of this study. Median parasite clearance half-lives in the three study groups were 3.54, 2.74, and 2.98 hours and there was no significant difference between the groups. Comparing these results to those from Dondorp *et al*. [3], median half-lives from the present study are again comparable to those observed in Wang Pha, western Thailand (3.0 and 2.7 hours) and much lower than those observed in Pailin (5.0 and 6.0 hours) in 2009.

Because parasite clearance after artemisinin treatment is rapid, most patients are expected to have cleared their peripheral parasitaemia by day 3 (72 hours) after the start of treatment. Thus, the proportion of positive blood smears on day 3 has been suggested as a tool for assessing population levels of artemisinin resistance. Data from more than 19,000 cases of acute *P*. *falciparum* malaria suggest that artemisinin resistance is highly unlikely if the proportion of patients with a positive smear on day 3 (72 hours) is <3% among patients with parasite densities of <100,000 parasites/μL at enrolment [9]. In this study, the proportion of patients with parasite clearance times >72 hours was >20% in all three study arms indicating an urgent need for more information.

In addition to the parasitaemia level on admission, there are other factors that influence parasite clearance such as age, individual immune status, types of anti-malarial drugs used (pharmacokinetic and pharmacodynamic properties), and parasite virulence. Recently, a study in western Cambodia suggested that some host factors, such as erythrocyte polymorphisms could affect parasite clearance half-life [21]. As mentioned previously, blood samples were collected from the patients of this study but results on host genetics could not be included in this manuscript. However, a large scale screening for hemoglobin disorders in southern Vietnam including Phuoc Long- Binh Phuoc area during 2003–2007 [22] showed that the gene frequency for haemoglobin E was 3.3% in Kinh and 44% in S’tieng ethnic people, the two largest communities in the study area. An exploratory analysis for the present study revealed no apparent or statistically significant difference between the parasite clearance half-lives of the two ethnic subgroups (data not shown).

Historical parasite clearance data from two comparable studies in southern Vietnam are summarized in Table 3. A randomized controlled clinical trial conducted between 2001 and 2002 at the Hospital for Tropical Diseases in Ho Chi Minh City, Vietnam, included patients with similar inclusions criteria to this study (parasitaemia > 10,000/μL and < 100,000 /μL) and treated them with artesunate (4 mg/kg single dose) *vs* artesunate (4 mg/kg on day 1, 2 mg/kg on days 2 and 3) both combined with mefloquine 15 mg/kg on day 3 *vs* a single dose of mefloquine 15 mg/kg. In that study, mean (range) parasite clearance times were 48.2 (40.6-55.9) hours and 44.5 (37.7-51.4) hours in the AS 2 mg/kg and AS 4 mg/kg groups, respectively, and no patients had positive blood smears at 72 hours [23]. A review of data from studies in Vietnam from 1998–2009 also reported a very low proportion of positive day 3 smears in patients receiving artemisinin (6% and 9%, in 1998 and 2001, respectively) or artesunate (4% and 2% in 2004/2005, 2008/2009, respectively) [24].

**Table 3:** **Parasite clearance time** (**PCT100**) **from studies 2000**–**2009 in Vietnam**

**(Hien TT 2004)**[23]	**Artesunate 1 day (4mg/kg) + mefloquine on day 3**		**Artesunate 3 days (4, 2, 2 mg/kg) + mefloquine on day 3**	
	**(N=70)**		**(N=73)**	
Parasite clearance time				
- Mean (range) hours	48.2 (40.6-55.9)		44.5 (37.7-51.4)	
(**Thanh NV et al**. **2010**) [24]	**1998**	**2001**	**2004**/**2005**	**2008**/**2009**
	(**Artemisinin 5 days**)	(**Artemisinin 7 days**)	(**Artesunate 7 days**)	(**Artesunate 7 days**)
	**N**=**65**	**N**=**69**	**N**=**82**	**N**=**54**
Parasite clearance time				
- Median (SD) days	1.8 ± 0.9	2.3 ± 0.9	2.1 ± 0.7	1.6 ± 0.7
Positive parasitaemia				
- On day 3 – n (%)	4 (6%)	6 (9%)	3 (4%)	1 (2%)
- On day 4 – n (%)	0	0	0	0

Given certain similarities between the epidemiological situation in southern Vietnam and Wang Pha, Thailand – namely, similar parasite clearance times and a decade-long pattern showing an increased proportion of slow clearers – it is imperative that detailed monitoring of the clinical phenotype of *P*. *falciparum* infections in Vietnam is performed. As the conclusions of the Thailand study [7] showed a genetically-attributable resistance phenotype evolving during this 10-year period, the possibility that artemisinin resistance has either evolved or been imported into southern Vietnam sometime during the past decade should be considered. Molecular studies currently being done will help determine the proportion of the clearance phenotype that can be explained by genetics, and ongoing clinical studies at multiple sites in Vietnam will provide data on changes in parasite clearance occurring between 2010 and 2012.

Efficacy comparisons between treatment groups were only a secondary aim of this study and it was only powered to detect large effects (>75% differences in parasite clearance half lives). Longer clearance half-lives were observed in the AS 2mg/kg group but the comparison of the primary endpoint between treatment groups did not reach statistical significance. However, there was a significantly higher risk of treatment failure (after PCR-correction) and a lower 24-hour parasite reduction ratio in the AS 2mg/kg group. This could be explained by the lower parasiticidal effect of the lower dose, possibly allowing a greater proportion of parasites to become “dormant” [25].

The impact of this study for malaria control in Vietnam is that health policy makers must begin considering whether the first-line drug combination (dihydroartemisinin-piperaquine) is the optimal choice to treat *falciparum* malaria in Vietnam. At the Thai-Myanmar border 10–20% of patients treated with ACT have been found to be parasitaemic after a three-day treatment [20] which, when considered alongside the >20% patients with positive smears on day 3 in this trial, suggests the geographic expansion of artemisinin drug resistance. The assessment of parasite clearance provided by frequent parasite counts in this study allows for a detailed characterization of therapeutic responses and has shown that maximum parasiticidal effects are probably not achieved by the 2mg/kg artesunate dose. However, overall responses to the currently recommended ACT remain adequate. Clearly, further close monitoring is required [20] to assess whether this represents the emergence of artemisinin resistance. However, for the present, the malaria control program in Vietnam still adheres to the dihydroartemisinin -piperaquine combination because there is no alternative anti-malarial drug which is as good, as available, or as affordable as the current one.

## Conclusions

This study demonstrates faster *P*. *falciparum* parasite clearance in southern Vietnam than in western Cambodia but slower clearance in comparison with historical data from Vietnam. Further studies to determine whether this represents the emergence of artemisinin resistance in this area are needed. Currently, the therapeutic response to DHA-PPQ remains satisfactory in southern Vietnam. Given the importance of current malaria research to informing global malaria eradication strategies, the response to artemisinin should be monitored in other regions of the world for the potential emergence and spread of artemisinin resistance, as artemisinin-based combination therapy is likely to be a key tool in helping to achieve and sustain malaria case reductions around the globe [26]. If artemisinin resistance does spread out of this region, new strategies and new techniques for resistance management and containment will be required [27][28] to maintain the case reductions that have been achieved over the past 10 years [29,30].

## Competing interests

The authors declare that they have no competing interests. PR is a staff member of the World Health Organization. The author alone is responsible for the views expressed in this publication and they do not necessarily represent the decisions, policy or views of the World Health Organization.

## Authors’ contribution

TTH, PR, NJW, MW, KS and JF contributed to the design of the study. TTH, NTTN, NHP, NVT, NTNC, LHT, CQT, PVT, PDT, CD, LTL, LTD and LM participated in recruitment of participants and data collection. MW and MFB performed the statistical analysis. TTH drafted the first version and all authors reviewed and approved for submission. All authors read and approved the final manuscript.

## Supplementary Material

Additional file 1 **Table S1.** Summary of primary and secondary endpoints by treatment group (per protocol population). Click here for file
