# Supplementary material for: In vivo susceptibility of Plasmodium falciparum to artesunate in Binh Phuoc Province, Vietnam
Source: Malar J. 2012 Oct 26;11:355. doi: 10.1186/1475-2875-11-355 (PMC3504531; doi:10.1186/1475-2875-11-355)
Supplement: Additional file 1 — Table S1. Summary of primary and secondary endpoints by treatment group (per protocol population). [file 1475-2875-11-355-S1.docx]

**Supplementary Table 1: Summary of primary and secondary endpoints by treatment group (per protocol population)**

| Characteristic | Artesunate 2mg/kg  (N=52) | Artesunate 4mg/kg  (N=54) | DHA-Piperaquine  (N=54) | Overall comparisons  (p-value) |
| --- | --- | --- | --- | --- |
| Clearance rate constant (“slope”) K  [ - log_e_ parasitemia/hour] $  - median (IQR) | 0.19(0.12,0.26) | 0.25(0.11,0.39) | 0.23(0.12,0.32) | 0.16 |
| Clearance half life T_1/2_ [hours]  - median (IQR) | 3.59(2.63,5.94) | 2.72(1.79,6.29) | 3.04(2.15,5.82) | 0.16 |
| Risk of failure (WHO)  [PCR-uncorrected]  - Kaplan-Meier analysis #  - Proportion (“per protocol”)  Risk of failure (WHO)  [PCR-corrected]  - Kaplan-Meier analysis #  - Proportion (“per protocol”) | 6%  3/49 (6%)  6%  3/49 (8%) | 0%  0/46 (0%)  0%  0/46 (0%) | 2%  1/52 (2%)  0%  0/51 (0%) | 0.16  0.21  0.04  0.07 |
| Parasite clearance time (PCT100)  - median (IQR) [hours] #  - Proportion with PCT>72h # | 60 (36, 78)  0.29 | 42 (30, 78)  0.26 | 48 (36, 72)  0.22 | 0.25  - |
| Parasite-reduction ratio (%) £  - at 24 hours – median (IQR)  - at 48 hours – median (IQR) | 40 (4,265)  2443 (137,Inf) | 212 (14,2493)  Inf (244,Inf) | 99 (12,556)  Inf (406,Inf) | 0.01  - |
| Fever clearance time #  - median (IQR) [hours] | 30 (18, 42) | 24 (12,42) | 24 (18,36) | 0.86 |

Summary measures in each group are median (IQR) or n (%).
($) Slope could not be estimated for one patient on Artesunate 4mg/kg, (#) Kaplan-Meier estimates, (£) Missing follow-up parasitemia values at 24 and 48 hours replaced by 0 if the patient had prior documented parasite clearance; no missing data remained after this imputation. Values of infinity (Inf) correspond to patients without parasitemia at 24 or 48 hours, respectively.
